# Panoramic Dental Radiography Image Enhancement Using Multiscale Mathematical Morphology

**DOI:** 10.3390/s21093110

**Published:** 2021-04-29

**Authors:** Julio César Mello Román, Vicente R. Fretes, Carlos G. Adorno, Ricardo Gariba Silva, José Luis Vázquez Noguera, Horacio Legal-Ayala, Jorge Daniel Mello-Román, Ricardo Daniel Escobar Torres, Jacques Facon

**Affiliations:** 1 Facultad Politécnica, Universidad Nacional de Asunción, San Lorenzo 111421, Paraguay; juliomello@pol.una.py (J.C.M.R.); hlegal@pol.una.py (H.L.-A.); 2 Facultad de Odontología, Universidad Nacional de Asunción, Asunción 001218, Paraguay; vicentefretes@odo.una.py (V.R.F.); cgadorno@odo.una.py (C.G.A.); 3 Departamento de Odontologia Restauradora, Faculdade de Odontologia de Ribeirão Preto, Universidade de São Paulo, Ribeirão Preto 14040-904, SP, Brazil; gariba@usp.br; 4 Facultad de Ciencias Exactas y Tecnológicas, Universidad Nacional de Conepción, Concepción 010123, Paraguay; jdmello@facet-unc.edu.py (J.D.M.-R.); dannytorres@facet-unc.edu.py (R.D.E.T.); 5 Department of Computer and Electronics, Universidade Federal do Espírito Santo, São Mateus 29932-540, ES, Brazil; jacques.facon@ufes.br

**Keywords:** panoramic dental radiography, contrast enhancement, multi-scale mathematical morphology, top-hat transformation

## Abstract

Panoramic dental radiography is one of the most used images of the different dental specialties. This radiography provides information about the anatomical structures of the teeth. The correct evaluation of these radiographs is associated with a good quality of the image obtained. In this study, 598 patients were consecutively selected to undergo dental panoramic radiography at the Department of Radiology of the Faculty of Dentistry, Universidad Nacional de Asunción. Contrast enhancement techniques are used to enhance the visual quality of panoramic dental radiographs. Specifically, this article presents a new algorithm for contrast, detail and edge enhancement of panoramic dental radiographs. The proposed algorithm is called *Multi-Scale Top-Hat transform powered by Geodesic Reconstruction for panoramic dental radiography enhancement* (MSTHGR). This algorithm is based on multi-scale mathematical morphology techniques. The proposal extracts multiple features of brightness and darkness, through the reconstruction of the marker (obtained by the Top-Hat transformation by reconstruction) starting from the mask (obtained by the classic Top-Hat transformation). The maximum characteristics of brightness and darkness are added to the dental panoramic radiography. In this way, the contrast, details and edges of the panoramic radiographs of teeth are improved. For the tests, MSTHGR was compared with the following algorithms: Geodesic Reconstruction Multiscale Morphology Contrast Enhancement (GRMMCE), Histogram Equalization (HE), Brightness Preserving Bi-Histogram Equalization (BBHE), Dual Sub-Image Histogram Equalization (DSIHE), Minimum Mean Brightness Error Bi-Histogram Equalization (MMBEBHE), Quadri-Histogram Equalization with Limited Contrast (QHELC), Contrast-Limited Adaptive Histogram Equalization (CLAHE) and Gamma Correction (GC). Experimentally, the numerical results show that the MSTHGR obtained the best results with respect to the Contrast Improvement Ratio (CIR), Entropy (E) and Spatial Frequency (SF) metrics. This indicates that the algorithm performs better local enhancements on panoramic radiographs, improving their details and edges.

## 1. Introduction

Panoramic dental radiography is one of the most widely used medical imaging techniques, both by the general practitioner and in the various dental specialties [1,2,3]. A good quality of the image obtained is essential to its correct interpretation. This can be affected by various factors such as the device used, the acquisition technique and the subsequent processing of the acquired images [4,5]. One of the limitations of this type of images is that it is a two-dimensional representation of a three-dimensional object, so the different anatomical structures are superposed in the images obtained.

Contrast enhancement techniques not only help the dentist make a better assessment of the radiographs, but they are also used as a preprocessing of other more advanced automatic identification schemes based on deep learning [6]. Many contrasts, brightness, detail and edge enhancement algorithms have been proposed. Histogram-based algorithms are very popular for improving image contrast, brightness and detail. Among the most popular are Histogram Equalization (HE) and Contrast-Limited Adaptive Histogram Equalization (CLAHE) [7]. There are many variants of the histogram-based algorithms, and they have been used to make improvements in different types of images [5,8,9,10,11,12,13,14,15]. Ahmad et al. [16] compared contrast enhancement techniques based on histograms. These techniques help to improve the visual quality of the images. This allows specialists to better identify pathologies within the images. In addition, Rahmi-Fajrin et al. [12] studied how contrast enhancement results affect the dentist’s evaluation. A relatively new contrast enhancement technique is based on mathematical morphology. This uses top-hat operations to extract bright and dark features from the image. These features are finally added to the original image. In this way, the contrast of the images is enhanced [17,18]. In [19], a method for improving chest radiographs that use Top-Hat transformations to iteratively extract useful image features is presented. The selection of the optimal scale is made taking into account the measurement of the contrast enhancement ratio. Other algorithms based on multi-scale mathematical morphology have been proposed more recently to improve the performance of Top-Hat transformations [20,21,22]. These schemes proved to be effective at improving contrast, detail and edges of images, and they have been applied to different types of images satisfactorily [20,21,22,23,24,25,26,27]. In [27], an algorithm using the Top-Hat transformations by reconstruction at multiple scales is presented. This makes it possible to improve the contrast of medical images without distorting them. There are also techniques with hybrid approaches, which consist of the strategic combination of two or more image enhancement techniques [4,28,29,30]. In [29], a hybrid contrast enhancement scheme based on CLAHE and morphological operations is presented. With this approach, it is possible to enhance contrast and reduce noise in dental X-ray images.

This article presents a novel contrast and detail enhancement algorithm for panoramic dental radiography images. The proposed enhancement approach uses multi-scale mathematical morphology. First, in each iteration, the classic top-hat transform (mask) of the image and the top-hat transform by image reconstruction (marker) are calculated. Then, the marker is reconstructed starting from the mask. Here, only the most important bright and dark anatomical structures that may be present in panoramic dental radiographs are taken into account. Next, the extracted features are fused together by calculating the maxima between all the iterations of the bright and dark scales. Finally, the most important bright and dark areas are added to the original image.

Experimental results were evaluated using metrics that quantify global contrast, local contrast and detail and edge enhancement. Then, dental specialists performed an objective evaluation of the results obtained by the algorithms in a blind way.

The most important contributions of the work are:

1. A novel algorithm for contrast, detail and edge enhancement of panoramic dental radiographs based on multi-scale mathematical morphology is proposed.
2. Objective clinical evaluation of the results, obtained by the algorithms, was performed by specialists.

The organization of the article is as follows. Section 2 presents the set of panoramic radiographs and the proposed algorithm. Section 3 presents the experiments, discussions and clinical evaluation of the results. Finally, Section 4 presents the conclusions of the work.

## 2. Materials and Methods

This section presents the dataset on which the tests are performed and the proposed algorithm.

### 2.1. Dataset

In total, 598 patients (361 female and 237 male) were selected from consecutive patients undergoing dental panoramic radiography at the Department of Radiology of the Faculty of Dentistry, Universidad Nacional de Asunción. Of these images, 107 images have the following characteristics: complete dentition, without restorations and without radiographically detectable pathologies. Images of this type were then used to make a clinical validation of the results obtained by the different algorithms to be compared in Section 3.4. The mean age was 36.7 years. Written informed consent was obtained from each patient following the school’s protocol. The digital radiograph images were stored in jpeg format with a resolution set to 2041 × 1024 pixels. The radiographs were taken between March 2015 and February 2020 using a panoramic radiograph (I-Max touch, Owandy Radiology, France) with a tube voltage of 80 kV, tube current of 9 mA and an acquisition time of 14.4 s. The dataset is available online https://doi.org/10.5281/zenodo.4457648, accessed on 26 January 2021.

**Figure 1:**
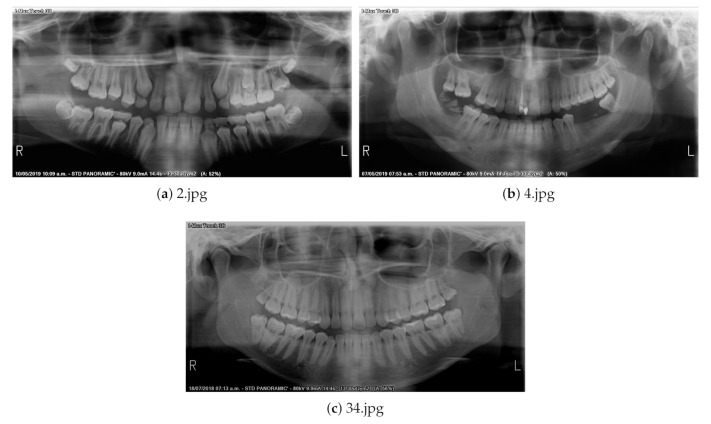
Example panoramic radiographs: (**a**) mixed dentition; (**b**) partially edentulous permanent dentition; and (**c**) complete permanent dentition.

The dataset contains mixed and permanent dentition images, with all teeth and missing teeth, no restorations and with different types of restorations. Example panoramic radiographs contained in the database are shown in Figure 1.

### 2.2. Proposed Algorithm

The proposed algorithm, described in this section, is called Multi-Scale Top-Hat transform powered by Geodesic Reconstruction for panoramic dental radiography enhancement (MSTHGR). The proposed algorithm (MSTHGR) is a variation of the method described by Roman et al. [27]. The main variation is in Equations (14) and (15). These equations seek to highlight the differences between the light and dark areas of the image (contrast) without altering too much the naturalness of the image. The results show images with greater detail and sharper edges.

Let *I* be the original image and *H* a flat structuring element. The dilation $\delta_{H}\left(I\right)$ and erosion $\varepsilon_{H}\left(I\right)$ are defined as follows [31]:

(1) $$\delta_{H}\left(I\right)\left(u,v\right)=\underset{(x,y)\in H}{max}\left(I\left(u-x,v-y\right)\right),$$

(2) $$\varepsilon_{H}\left(I\right)\left(u,v\right)=\underset{(x,y)\in H}{min}\left(I\left(u+x,v+y\right)\right),$$

where (u,v) and (x,y) are the pixel coordinates of *I* and *H*, respectively. The opening and closing operations can be defined from the dilation and erosion operations.

Morphological opening [17] is obtained by dilating the image that is first eroded using the same structuring element. Morphological opening of *I* for a *H* structuring element is denoted by γ(I,H) and defined as:

(3) $$\gamma \left(I,H\right)=\delta_{\overset{ˇ}{H}}\left(\varepsilon_{H}\left(I\right)\right),$$

where $\overset{ˇ}{H}$ is the reflection of *H*.

Morphological closing [17] is obtained by eroding the image that is first dilated using the same structuring element. Morphological closing of *I* for a *H* structuring element is denoted by ϕ(I,H) and defined as:

(4) $$\phi \left(I,H\right)=\varepsilon_{\overset{ˇ}{H}}\left(\delta_{H}\left(I\right)\right).$$

If a structuring element is symmetrical, then it is equal to its reflection, i.e., $H=\overset{ˇ}{H}$.

Top-hat transform by opening (WTH) [17] is obtained by subtracting from image *I* its morphological opening γ(I,H), and it is defined as follows:

(5) WTH(I,H)=I−γ(I,H).

Top-hat transform by closing (BTH) [17] is obtained by subtracting from the morphological closing ϕ(I,H) the image *I*, and it is defined as follows:

(6) BTH(I,H)=ϕ(I,H)−I.

In geodesic transformations, two input images are used. The first image is modified by a morphological transformation and restricted above or below the second image. The morphological transformations allowed are erosion and dilation [32].

Let *g* be the marker image and *I* be the mask image and both have the same domain ($D_{g}$ = $D_{I}$ and g≤I). The geodesic dilation of size 1 or $\delta_{I}^{\left(1\right)}\left(g\right)$ is defined as the minimum point to point between *I* and the dilation of *g*, i.e., $\delta_{I}^{\left(1\right)}\left(g\right)=\min\left(\delta_{H}\left(g\right),I\right)$. If we perform *m* times the geodesic dilation of *g* with respect to *I*, we have to $\delta_{I}^{\left(m\right)}\left(g\right)=\delta_{I}^{\left(1\right)}\left(g\right)\left[\delta_{I}^{\left(m-1\right)}\left(g\right)\right]$. Geodesic dilation of *g* from *I* iterated to stability is called dilation by reconstruction [32] and is defined as:

(7) $$R_{I}^{\delta }\left(g\right)=\delta_{I}^{\left(s\right)}\left(g\right),$$

where *s* is such that $\delta_{I}^{\left(s\right)}\left(g\right)=\delta_{I}^{\left(s+1\right)}\left(g\right)$.

Let *g* be the marker image and *I* be the mask image and both have the same domain ($D_{g}$ = $D_{I}$ and g≥I).

The geodesic erosion of size 1 or $\varepsilon_{I}^{\left(1\right)}\left(g\right)$ is defined as the maximum point to point between *I* and the erosion of *g*, i.e., $\varepsilon_{I}^{\left(1\right)}\left(g\right)=\max\left(\varepsilon_{H}\left(g\right),I\right)$. If we perform *m* times the geodesic erosion of *g* with respect to *I* we have to $\varepsilon_{I}^{\left(m\right)}\left(g\right)=\varepsilon_{I}^{\left(1\right)}\left(g\right)\left[\varepsilon_{I}^{\left(m-1\right)}\left(g\right)\right]$.

Geodesic erosion of *g* from *I* iterated to stability is called erosion by reconstruction [32] and is defined as:

(8) $$R_{I}^{\varepsilon }\left(g\right)=\varepsilon_{I}^{\left(s\right)}\left(g\right),$$

where *s* is such that $\varepsilon_{I}^{\left(s\right)}\left(g\right)=\varepsilon_{I}^{\left(s+1\right)}\left(g\right)$.

Opening by reconstruction [32] of an *I* image is defined as the reconstruction of *I* from the *m* size erosion of *I*:

(9) $$\gamma_{R}^{\left(m\right)}\left(I\right)=R_{I}^{\delta }\left(\varepsilon_{H_{m}}\left(I\right)\right),$$

where *I* is the mask image, $\varepsilon_{H_{m}}\left(I\right)$ is the marker image and *m* is a scale factor of the structuring element. If $H_{m}$ is convex, then it is obtained as follows:

(10) $$H_{m}=\underset{\mathrm{dilation}\;m-1\;\mathrm{times}}{\underset{︸}{H_{1}⊕H_{1}⊕\cdots ⊕H_{1}}},$$

where ⊕ is the operator of binary dilation. Binary dilation is the sum (vector) of all possible pairs of coordinate points of the $H_{1}$ set. That is, let *p* and *q* be the spatial coordinates of $H_{1}$, then $H_{1}⊕H_{1}=\left\{\left(p+q\right)|\;\mathrm{for}\;\mathrm{all}\;p,q\in H_{1}\right\}$.

Closing by reconstruction [32] of an *I* image is defined as the reconstruction of *I* from the *m* size dilation of *I*:

(11) $$\phi_{R}^{\left(m\right)}\left(I\right)=R_{I}^{\varepsilon }\left(\delta_{H_{m}}\left(I\right)\right),$$

where *I* is the mask image and $\delta_{H_{m}}\left(I\right)$ is the marker image.

Structures removed by the opening by reconstruction can be recovered by the white top-hat transform by Reconstruction (RWTH) [32]:

(12) $$RWTH\left(I\right)=I-\gamma_{R}\left(I\right).$$

Similarly, structures removed by the closing by reconstruction can be recovered by the dark top-hat Transformation by Reconstruction (RBTH) [32]:

(13) $$RBTH\left(I\right)=\phi_{R}\left(I\right)-I.$$

The multiple features of the bright areas are obtained, which are extracted by RTH [33] as follows:

(14) $$RTH_{i}=R_{WT_{i}}^{\delta }\left(RW_{i}\right),$$

where $WT_{i}=WTH_{i}\left(I,H_{i}\right)$ is the mask image obtained by Equation (5), $H_{i}=\underset{\mathrm{dilation}\;i-1\;\mathrm{times}}{\underset{︸}{H_{1}⊕H_{1}⊕\cdots ⊕H_{1}}}$ (⊕ is the operator of binary dilation) and 1≤i≤n, $RW_{i}=RWTH_{i}\left(I\right)=I-\gamma_{R}^{\left(i\right)}\left(I\right)$ is the marker image obtained by Equation (12), $RTH_{i}$ are the *i*-scales of brightness that are extracted from the image and i={1,2,3,...,n}.

Similarly, the multiple features of the dark areas of the image are extracted using RBH [33] as follows:

(15) $$RBH_{i}=R_{BT_{i}}^{\delta }\left(RB_{i}\right),$$

where $BT_{i}=BTH_{i}\left(I,H_{i}\right)$ is the mask image obtained by Equation (6), $RB_{i}=RBTH_{i}\left(I\right)=\phi_{R}^{\left(i\right)}\left(I\right)-I$ is the marker image obtained by Equation (13) and $RBH_{i}$ are the *i* scales of darkness extracted from the image.

The subtractions between the multiple scales of the bright regions of the image are obtained as follows [27]:

(16) $$SW_{i-1}=RTH_{i}-RTH_{i-1}.$$

Similarly, the subtractions between the multiple scales of the dark regions of the image are obtained as follows [27]:

(17) $$SB_{i-1}=RBH_{i}-RBH_{i-1}.$$

The maximum is obtained from all the multiple scales obtained in the different stages. The maximum bright and dark scales, extracted from the image, are calculated as follows:

(18) $$SRTH=\max_{1\leq i\leq n}\left\{RTH_{i}\right\},$$

(19) $$SRBH=\max_{1\leq i\leq n}\left\{RBH_{i}\right\}.$$

**Figure 2:**
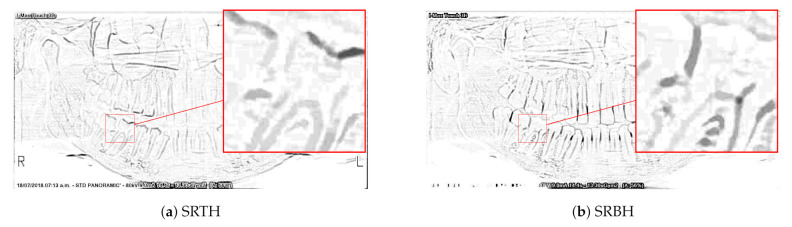
Bright and dark areas (maximum).

Figure 2 shows the bright and dark areas (maximum) extracted at the multiple scales. The pixels in Figure 2a,b were multiplied by a constant equal to 10, and then their complement was calculated. This is to better highlight the contour of the teeth. These operations were made for better visualization by the reader and they are not real operations applied to the algorithm.

All maximum bright and dark scales, extracted from the image by subtraction, are obtained as follows:

(20) $$SSW=\max_{2\leq i\leq n}\left\{SW_{i-1}\right\},$$

(21) $$SSB=\max_{2\leq i\leq n}\left\{SB_{i-1}\right\}.$$

**Figure 3:**
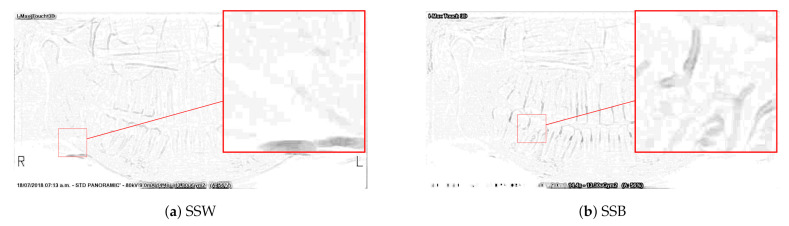
Differences in the bright and dark areas (maximum).

Figure 3 shows the maximum of the differences in the bright and dark areas. The pixels in Figure 3a,b were multiplied by a constant equal to 10, and then their complement was calculated. This is to better highlight the contour of the teeth. These operations were made for better visualization by the reader and they are not real operations applied to the algorithm.

Finally, image enhancement is carried out as follows:

(22) $$I_{E}=I+\left(SRTH+SSW\right)-\left(SRBH+SSB\right),$$

where $I_{E}$ is the panoramic dental radiography with contrast enhancement.

**Figure 4:**
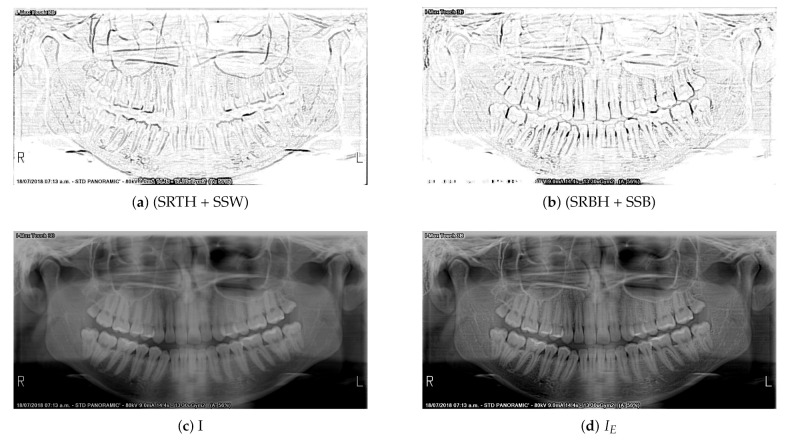
Enhancement of panoramic radiograph 34.jpg.

Figure 4 shows the final process of enhancing panoramic radiographs. The pixels in Figure 4a,b were multiplied by a constant equal to 10, and then their complement was calculated. This is to better highlight the contour of the teeth. These operations were made for better visualization by the reader and they are not real operations applied to the algorithm.

### 2.3. Edge Detection or Segmentation Application

**Figure 5:**
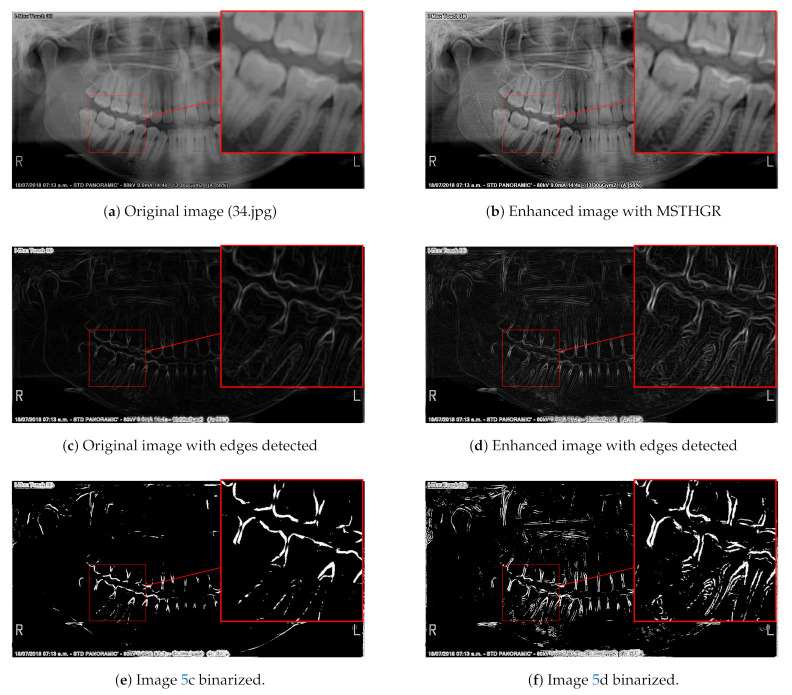
Application of an edge detection algorithm on an image preprocessed with MSTHGR.

Image enhancement algorithms are commonly used to adjust images, either for visual interpretation or as preprocessing for other algorithms such as edge detection or segmentation. The image features extracted by segmentation or edge detection algorithms are then used by classifiers to determine to which group the observed object belongs. An application that consists of first applying an edge detection (for example Sobel’s algorithm) to the panoramic radiograph and then a thresholding algorithm to decompose it into its most representative parts can be seen in Figure 5. The Sobel algorithm performed better edge detection in the enhanced image and its thresholding (algorithm based on maximum entropy [34]) shows more representative parts of the image, as can be seen in Figure 5d,f.

Another potential utility of enhanced panoramic radiographs could be their use in a learning environment, for example teaching and practicing interpretation of normal anatomical structures in panoramic radiographs.

## 3. Results and Discussion

This section presents the experimental results carried out to validate the proposed algorithm. To successfully validate the proposed algorithm, two objectives were set:

- To quantify the performance of the proposed algorithm in terms of improving panoramic dental radiography. For this purpose, comparisons were made against other state-of-the-art algorithms and evaluation metrics were used to quantify the numerical results obtained by the algorithms.
- Analyze clinically in an objective way how contrast enhancement algorithms affect panoramic radiographs. For this purpose, dentists performed a visual evaluation and objectively assess a sample of the results obtained.

### 3.1. Assessment Metrics

The results were evaluated with the following metrics:

- Relative Enhancement in Contrast (*REC*) [35,36] quantifies the contrast of the enhanced panoramic radiography. The greater the *REC* is, the better contrast the dental image will have. *REC* is defined as,
  (23) $$REC=\frac{C(I_{E})}{C(I)},$$
  where *I* is panoramic dental radiography, $I_{E}$ is dental imaging with contrast enhancement and *C* is image contrast. *C* is defined as,
  (24) $$C\left(I\right)=20\times \log\left[\frac{1}{MN}\sum_{u=1}^{M}\sum_{v=1}^{N}\left({\left(I\left(u,v\right)\right)}^{2}-{\left(\frac{1}{MN}\sum_{u=1}^{M}\sum_{v=1}^{N}I\left(u,v\right)\right)}^{2}\right)\right],$$
  where M×N are the dimensions of the dental image and (u,v) are the spatial coordinates.
- Contrast Improvement Ratio (*CIR*) [37] quantifies the local contrast of the enhanced medical image. The greater the *CIR* is, the better local contrast the medical image will have. *CIR* is defined as,
  (25) $$CIR\left(I,I_{E}\right)=\frac{\sum_{(u,v)\in D}|\omega \left(u,v\right)-\tilde{\omega }{\left(u,v\right)|}^{2}}{\sum_{(u,v)\in D}\omega {\left(u,v\right)}^{2}},$$
  where ω is local contrast of original dental imaging, $\tilde{\omega }$ is local contrast of enhanced dental imaging and *D* is the domain of values. ω is defined as,
  (26) $$\omega \left(u,v\right)=\frac{\left|\rho -\iota \right|}{\left|\rho +\iota \right|},$$
  where ρ is center pixel and ι is the average of the neighboring values in a window of 3×3.
- Entropy (*E*) [11,24,36], in digital image processing, is used to quantify the details or features of the image. The greater is the *E*, the better is the detail. E is defined as,
  (27) $$E\left(I\right)=-\sum_{k=0}^{L-1}P\left(k\right)log_{2}\left(P\left(k\right)\right),$$
  where *k* is intensity of the pixel in the image, P(k) is probability of occurrence of the k-value in the image, *b* is number of bits of the image and *L* is equal to $2^{b}$ and b=8 for grayscale images.
- Spatial Frequency (*SF*) [38], in digital image processing, is the metric that quantifies the spatial information contained in the image. If SF has a large value, the enhanced panoramic radiograph is considered to have more spatial information. SF is defined as follows:
  (28) $$SF=\sqrt{RF^{2}+CF^{2}},$$
  where
  (29) $$RF=\sqrt{\frac{1}{M\times N}\sum_{u=1}^{M}\sum_{v=1}^{N}{\left[I\left(u,v\right)-I\left(u-1,v\right)\right]}^{2}},$$
  (30) $$CF=\sqrt{\frac{1}{M\times N}\sum_{u=1}^{M}\sum_{v=1}^{N}{\left[I\left(u,v\right)-I\left(u,v-1\right)\right]}^{2}}.$$
- Peak signal-to-noise ratio (*PSNR*) [22,27], in digital image processing, is the metric adopted to quantify the distortion introduced in the image enhancement process. If PSNR has a large value, the enhanced panoramic radiograph is considered to have less distortion. PSNR is defined as follows:
  (31) $$PSNR\left(I,I_{E}\right)=10\times log_{10}\frac{{\left(L-1\right)}^{2}}{MSE(I,I_{E})}.$$
  The *Mean Squared Error* (*MSE*) is defined as:
  (32) $$MSE\left(I,I_{E}\right)=\frac{1}{M\times N}\sum_{u=0}^{M-1}\sum_{v=0}^{N-1}{\left(I\left(u,v\right)-I_{EN}\left(u,v\right)\right)}^{2}.$$
- Absolute Mean Brightness Error (*AMBE*) [22], in digital image processing, is the metric that quantifies the average brightness preservation of enhanced panoramic radiographs. If AMBE has a small value, the enhanced panoramic radiograph is considered to have preserved its average brightness. The AMBE is defined as follows:
  (33) $$AMBE\left(I,I_{E}\right)=\left|A\left(I\right)-A\left(I_{E}\right)\right|,$$
  where A(I) is the average brightness of the panoramic radiographs and $A(I_{E})$ is the average brightness of the panoramic radiographs with contrast enhancement.

### 3.2. Comparator Algorithms

MSTHGR was compared with the following algorithms: Geodesic Reconstruction Multiscale Morphology Contrast Enhancement (GRMMCE) [27], Histogram Equalization (HE), Brightness Preserving Bi-Histogram Equalization (BBHE) [8], Dual Sub-Image Histogram Equalization (DSIHE) [9], Minimun Mean Brightness Error Bi-Histogram Equalization (MMBEBHE) [10], Quadri-Histogram Equalization with Limited Contrast (QHELC) [13], Contrast-Limited Adaptive Histogram Equalization (CLAHE) [7] and Gamma Correction (GC) [39].

The MSTHGR, GRMMCE, HE, BBHE, DSIHE, MMBEBHE and QHELC algorithms were implemented using the ImageJ library [40]; for MSTHGR and GRMMCE, based on mathematical morphology, an extra library called MorphoLibJ [41] was used.

The MSTHGR and GRMMCE algorithms have the following parameters: original image *I*, number of iterations n=7 and a disk shape structuring element with initial radius r=1, where the radius increases in each iteration in a range r={1,2,3,⋯,n}. Bai [42] used a small number of iterations, because the best details that can be extracted from the image are at small scales. Therefore, in this experiment, we used the number of iterations n=7.

The CLAHE and GC algorithms were implemented in MATLAB 2014b. The parameters used were the default ones.

### 3.3. Numerical and Visual Results

The algorithms were tested on the 598 panoramic radiographs. Table 1 shows the average numerical results obtained by the algorithms. The two best average results are highlighted in bold. The MSTHGR algorithm obtained high average results in the CIR, E and SF metrics. However, it is also competitive in the other metrics.

**Figure 6:**
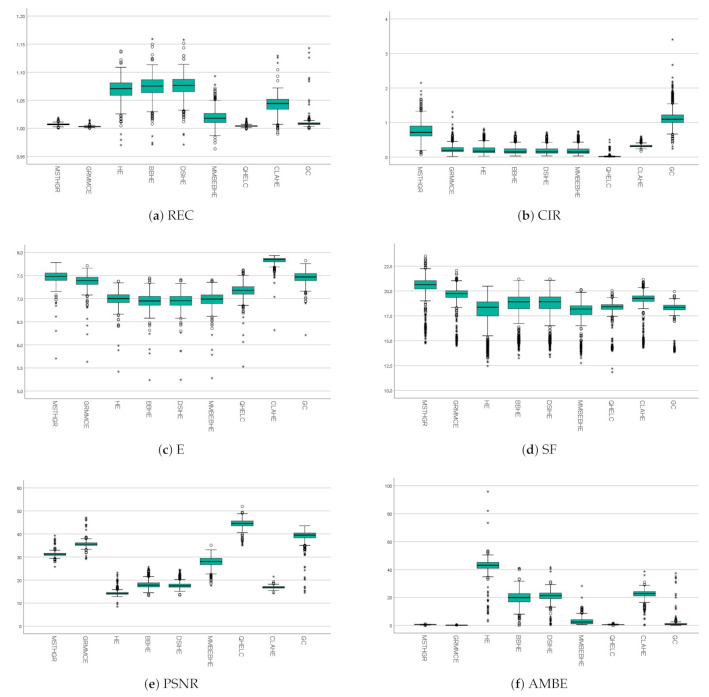
Box plots of the results obtained by the algorithms.

The proposed MSTHGR method is slightly better than GRMMCE (REC) in terms of contrast, but it is worse in terms of noise (PSNR) and Absolute Mean Brightness Error (AMBE). This is because the contrast enhancement is in trade-off relation to noise and average brightness. The better is the contrast values (REC), the worse are the PSNR and AMBE values. This can be seen in Figure 6. However, the improvement obtained by the MSTHGR method achieves better clinical assessments by dentists (this can be seen in Section 3.4).

Figure 6 shows the results of each metric in box plots. It can be observed that MSTHGR obtained less dispersed numerical values for the E and AMBE metrics. The values obtained by MSTHGR in the E, PSNR and SF metrics had a similar dispersion to the set of algorithms that integrate this study. It is also possible to observe that MSTHGR obtained values for the CIR metric with a similar dispersion to GC, but relatively higher than that obtained by the other algorithms.

The statistical analysis was carried out by means of non-parametric tests considering the amount of data in the database. Paired observations were made through the Wilcoxon Signed Rank Test [43], taking as pairs the results of each algorithm with respect to the proposed algorithm. The results of the Wilcoxon Signed Rank Test are presented in Table 2.

With respect to the evaluated database and at a statistical significance level of α = 0.01, the following can be observed:

- For the REC metric, MSTHGR was numerically superior to the GRMMCE, QHELC and GC algorithms.
- For the CIR metric, MSTHGR was numerically superior to the GRMMCE, HE, BBHE, DSIHE, MMBEBHE, QHELC and CLAHE algorithms.
- For the E metric, MSTHGR was numerically superior to the GRMMCE, HE, BBHE, DSIHE, MMBEBHE, QHELC and GC algorithms.
- For the SF metric, MSTHGR was numerically superior to all compared algorithms.
- For the PSNR metric, MSTHGR was numerically superior to the HE, BBHE, DSIHE, MMBEBHE and CLAHE algorithms.
- For the AMBE metric, MSTHGR was numerically superior to the HE, BBHE, DSIHE, MMBEBHE, CLAHE and GC algorithms.

The *p*-value close to zero (≈0) in the Wilcoxon signed-rank test for most of the paired observations leads to the conclusion that there are statistically significant differences between the medians obtained by the proposed MSTHGR algorithm and the other algorithms.

**Figure 7:**
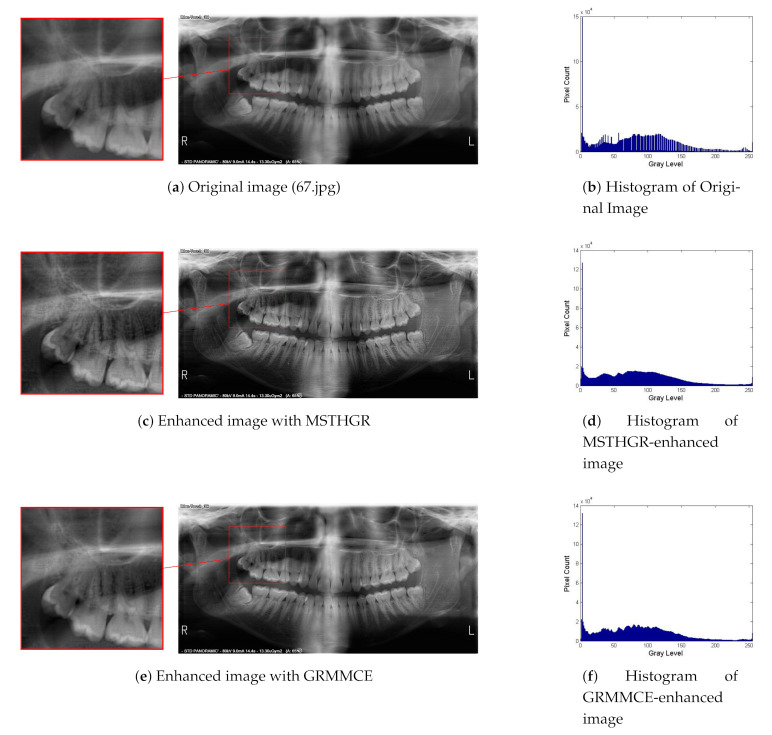

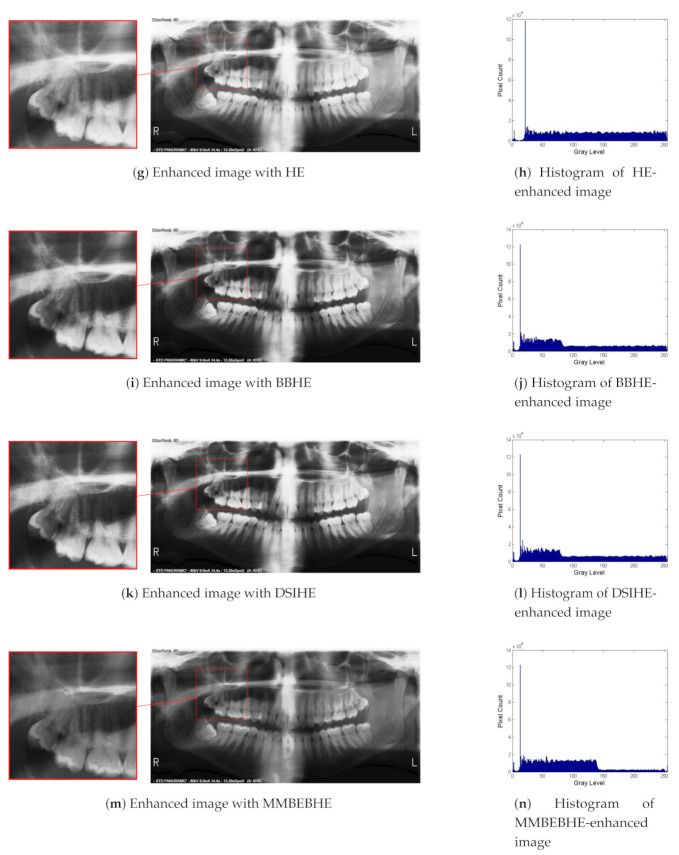

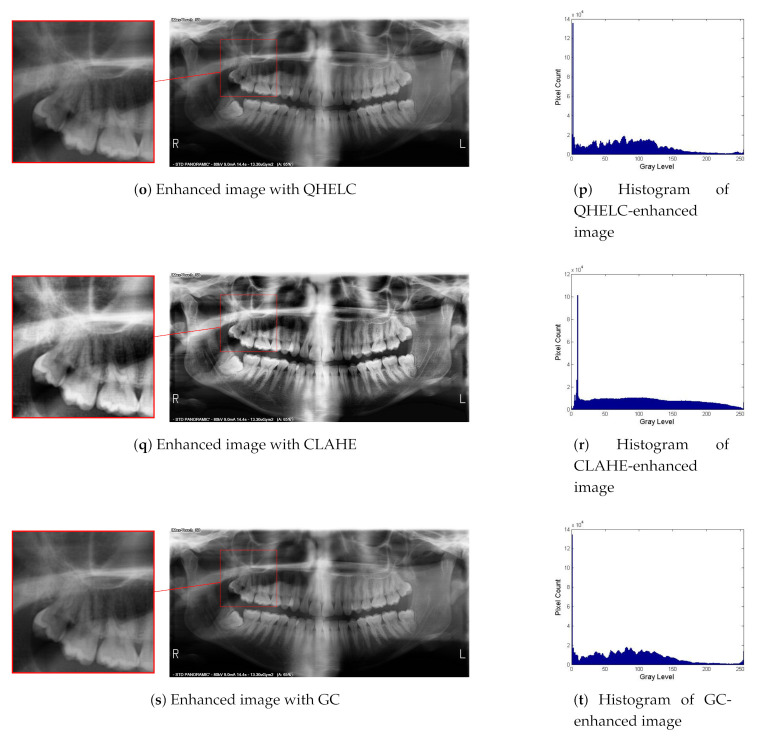
Visual results.

Figure 7 shows that the panoramic dental radiographs, enhanced with the MSTHGR algorithm, presented a greater definition at the edges of the teeth in general, as well as a better visualization of the different structures, such as enamel, dentin and pulp chamber, which compose them. In addition, they allowed a visualization of the restorations at the coronary level in the cases where they were present. At the root level, the light of the root canals could be observed with greater definition. At the level of the mandibular condyle, a better definition of the edges could be observed as well as a better observation of the bone trabeculae. In the regions of the maxillary sinuses and inferior dental canal, no great variations were found in comparison with the original images.

### 3.4. Clinical Validation and Prospects

The objective of the clinical validation is to quantify how the enhancement algorithms affect panoramic radiographs from the perspective of the dentists. In other words, the aim is to check whether the results obtained in the previous experiment are related to those observed by the dentists. For this purpose, 20 sample images were first taken from a total of 107 images with the following characteristics: complete dentition, without restorations and without radiographically detectable pathologies. This represents a sample with a 22% error rate and a 99% confidence rate. Next, these 20 images were processed with the MSTHGR, GRMMCE, HE, BBHE, DSIHE, MMBEBHE and QHELC algorithms and presented to a group of three dental specialists for qualitative evaluation. The algorithms were anonymized by a numerical code as follows: BBHE (1); DSIHE (2); HE (3); MMBEBHE (4); MSTHGR (5); GRMMCE (6); QHELC (7); CLAHE (8); and GC (9). The dentists performed an evaluation of the improved radiographs with respect to the original image. The observation was made in seven different anatomical regions: maxillary anterior teeth, maxillary posterior teeth, maxillary sinuses, mandibular anterior teeth, mandibular posterior teeth, mandibular condyles and mandibular canals. For each region, the scores were: 0 (worse than the original), 1 (no variation) and 2 (better than the original). Therefore, 9 algorithms × 20 images × 7 regions were analyzed, making a total of 1260 regions analyzed.

#### 3.4.1. Statistical Analysis

The sum of scores obtained in the seven anatomical regions for each enhancement/algorithm was the dependent variable treated as numerical data. Interobserver agreement was determined by the intraclass correlation coefficient (ICC). The criteria for strength of agreement proposed by Koo and Lee [44] were used for ICC interpretation: 0.00–<0.50, poor agreement; 0.5–0.75, moderate agreement; >0.75–0.90, good agreement; and >0.90, excellent agreement. Normality (Shapiro–Wilk) and Homogeneity of Variance (Levene) tests were statistically significant (*p* < 0.05). Therefore, a Kruskal–Wallis test was run to determine if there were differences in median sum of scores according to enhancement/algorithm. Statistical analysis and graphical representation of data were performed with R version 3.6.0 at a 5% significance level.

#### 3.4.2. Descriptive Statistics

Interobserver agreement was good (ICC = 0.835, 95% CI: 0.779–0.877). The Kruskall–Wallis test was statistically significant (*p* < 0.001). The results of the multiple comparisons using the Dunn test with Benjamini–Hochberg adjustment are shown in Table 3 (first column).

**Figure 8:**
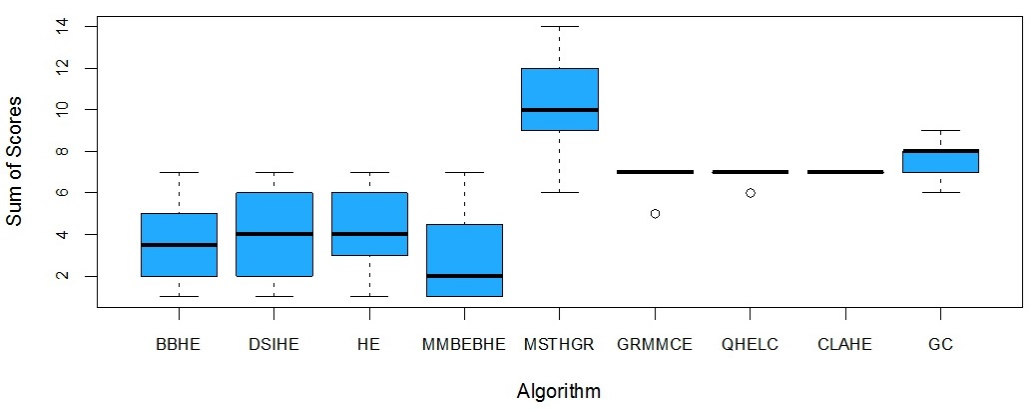
Box and whiskers diagram. Distribution of the sum of scores according to the enhancement algorithm used.

In the box and whisker plot (Figure 8), the medians are marked with a horizontal line in the box. The lower and upper limits of the box are the first (Q${}_{1}$) and third (Q${}_{3}$) quartiles, respectively. Values below Q${}_{1}$ and over Q${}_{3}$ are located in the whiskers. Outliers are represented by open dots.

In general, we can conclude that the algorithm MSTHGR improves the visual quality of the images with respect to the compared algorithms.

### 3.5. Usefulness for Diagnosis in Clinical Settings

The proposed algorithm allowed a clear observation of the teeth as well as the other anatomical regions analyzed in the selected images. Moreover, the different dental hard tissues (enamel–dentin junction), root canals and periodontal ligament were clearly visible.

## 4. Conclusions

Contrast and detail enhancement of panoramic radiographs helps dentists better evaluate the anatomical structures of the teeth present in the image. For this reason, an algorithm for contrast and detail improvement of panoramic dental radiographs is presented in this work. In addition, the help of dental specialists in the quantification of the results obtained by the algorithms is highlighted. They objectively evaluated the real impact of the improvements and how they affected the panoramic dental radiographs.

Experimentally, the numerical results show that the MSTHGR obtained the best results with respect to the CIR, E and SF metrics. This indicates that the proposal makes local improvements to the panoramic radiographs, enhancing their details and edges. With respect to the REC, PSNR and AMBE metrics, the proposal improves the global contrast of the radiographs, introduces less distortion to the image and preserves the average brightness. Even though its numerical results are inferior to some algorithms, the proposed method proved to be competitive.

The visual results obtained by the algorithms were clinically evaluated by specialist dentists. In general, according to the evaluations, the MSTHGR algorithm was better at enhancing the visual quality of panoramic dental radiographs.

As future work, quantitative evaluations of the improvements made to the images by areas of interest can be performed. In addition, this algorithm can be used as a preprocessing of other applications for automatic detection of pathologies affecting bone (cysts and tumors) or teeth (resorptions and caries).

## Figures and Tables

**Table 1:** Average results obtained by the algorithms.

Algorithms	REC	CIR	E	SF	PSNR	AMBE
I	-	-	7.209	17.681	-	-
MSTHGR	1.007	**0.773**	**7.462**	**20.019**	31.242	0.548
GRMMCE	1.003	0.245	7.369	**19.051**	35.700	**0.103**
HE	1.069	0.220	6.989	17.802	14.437	42.027
BBHE	**1.074**	0.197	6.943	18.314	18.147	19.601
DSIHE	**1.075**	0.197	6.942	18.327	17.801	20.741
MMBEBHE	1.019	0.194	6.975	17.588	27.787	3.081
QHELC	1.004	0.029	7.169	17.745	**44.536**	**0.529**
CLAHE	1.042	0.335	**7.820**	18.590	16.857	22.288
GC	1.010	**1.151**	7.458	17.705	**38.690**	1.508

**Table 2:** The Wilcoxon Signed Rank Test for paired observations.

Algorithms		Metrics					
		REC	CIR	E	SF	PSNR	AMBE
MSTHGR—GRMMCE	Negative ranks	0	0	4	0	598	4
	Positive ranks	598	598	594	598	0	594
	Z	−21.187	−21.187	−21.184	−24.413	−24.413	−24.086
	Sig. asymptotic (bilateral)	≈0	≈0	≈0	≈0	≈0	≈0
MSTHGR—HE	Negative ranks	595	20	0	0	0	598
	Positive ranks	3	578	598	598	598	0
	Z	−21.171	−20.85	−21.187	−24.413	−24.413	−24.413
	Sig. asymptotic (bilateral)	≈0	≈0	≈0	≈0	≈0	≈0
MSTHGR—BBHE	Negative ranks	595	20	0	1	0	597
	Positive ranks	3	578	598	597	598	1
	Z	−21.171	−20.924	−21.187	−24.331	−24.413	−24.331
	Sig. asymptotic (bilateral)	≈0	≈0	≈0	≈0	≈0	≈0
MSTHGR—DSIHE	Negative ranks	595	20	0	1	0	598
	Positive ranks	3	578	598	597	598	0
	Z	−21.178	−20.935	−21.187	−24.331	−24.413	−24.413
	Sig. asymptotic (bilateral)	≈0	≈0	≈0	≈0	≈0	≈0
MSTHGR—MMBEBHE	Negative ranks	499	21	0	0	32	595
	Positive ranks	99	577	598	598	566	3
	Z	−17.355	−20.922	−21.187	−24.413	−21.796	−24.168
	Sig. asymptotic (bilateral)	≈0	≈0	≈0	≈0	≈0	≈0
MSTHGR—QHELC	Negative ranks	35	11	0	0	598	268
	Positive ranks	563	587	598	598	0	330
	Z	−19.734	−21.159	−21.187	−24.413	−24.413	−2.494
	Sig. asymptotic (bilateral)	≈0	≈0	≈0	≈0	≈0	0.013
MSTHGR—CLAHE	Negative ranks	589	26	598	0	0	596
	Positive ranks	9	572	0	598	598	2
	Z	−23.677	−22.287	−24.413	−24.413	−24.413	−24.250
	Sig. asymptotic (bilateral)	≈0	≈0	≈0	≈0	≈0	≈0
MSTHGR—GC	Negative ranks	416	566	167	4	580	408
	Positive ranks	182	32	431	594	18	190
	Z	−9.528	−21.796	−10.755	−24.086	−22.941	−8.874
	Sig. asymptotic (bilateral)	≈0	≈0	≈0	≈0	≈0	≈0

**Table 3:** Descriptive statistics of the sum of scores according to the enhancement algorithm applied.

Algorithm	Sample	Average	Standard Deviation	Minimum	$Q_{1}$	Median	$Q_{3}$	Maximum
BBHE ${}^{a}$	20	3.60	1.789	1	2	3.5	4.50	7
DSIHE ${}^{a}$	20	3.85	1.843	1	2	4.0	6.00	7
HE ${}^{a}$	20	4.25	1.713	1	3	4.0	6.00	7
MMBEBHE ${}^{a}$	20	3.00	2.200	1	1	2.0	4.25	7
MSTHGR ${}^{c}$	20	10.00	2.077	6	9	10.0	12.00	14
GRMMCE ${}^{b}$	20	6.80	0.616	5	7	7.0	7.00	7
QHELC ${}^{b}$	20	6.90	0.308	6	7	7.0	7.00	7
CLAHE ${}^{b}$	20	7.00	0.000	7	7	7.0	7.00	7
GC ${}^{bc}$	20	7.55	0.826	6	7	8.0	8.00	9

**Note**: Different superscript letters in the first column indicate statistically significant differences (*p* < 0.05) according to Dunn test with Benjamini–Hochberg adjustment.

## Data Availability

The data presented in this study are available on request from the corresponding author.
